# Mitochondrial glutamine metabolism regulates sensitivity of cancer cells after chemotherapy via amphiregulin

**DOI:** 10.1038/s41420-021-00792-7

**Published:** 2021-12-20

**Authors:** Sunsook Hwang, Seungyeon Yang, Minjoong Kim, Youlim Hong, Byungjoo Kim, Eun Kyung Lee, Seung Min Jeong

**Affiliations:** 1 Department of Biochemistry, College of Medicine, The Catholic University of Korea, 222, Banpo-daero, Seocho-gu, Seoul, 06591 Republic of Korea; 2 Institute for Aging and Metabolic Diseases, College of Medicine, The Catholic University of Korea, 222, Banpo-daero, Seocho-gu, Seoul, 06591 Republic of Korea; 3 Department of Biomedicine & Health Sciences, College of Medicine, The Catholic University of Korea, 222, Banpo-daero, Seocho-gu, Seoul, 06591 Republic of Korea

**Keywords:** Cancer metabolism, Chemotherapy

## Abstract

The DNA damage response is essential for sustaining genomic stability and preventing tumorigenesis. However, the fundamental question about the cellular metabolic response to DNA damage remains largely unknown, impeding the development of metabolic interventions that might prevent or treat cancer. Recently, it has been reported that there is a link between cell metabolism and DNA damage response, by repression of glutamine (Gln) entry into mitochondria to support cell cycle arrest and DNA repair. Here, we show that mitochondrial Gln metabolism is a crucial regulator of DNA damage-induced cell death. Mechanistically, inhibition of glutaminase (GLS), the first enzyme for Gln anaplerosis, sensitizes cancer cells to DNA damage by inducing amphiregulin (AREG) that promotes apoptotic cell death. GLS inhibition increases reactive oxygen species production, leading to transcriptional activation of AREG through Max-like protein X (MLX) transcription factor. Moreover, suppression of mitochondrial Gln metabolism results in markedly increased cell death after chemotherapy in vitro and in vivo. The essentiality of this molecular pathway in DNA damage-induced cell death may provide novel metabolic interventions for cancer therapy.

## Introduction

Cells encounter many internal and external DNA damages. To respond to these threats, cells have evolved a tightly orchestrated signaling response from the activation of growth arrest or DNA repair pathways to the initiation of cell death, and defects in these well-coordinated DNA damage responses are frequent causes of genomic instability and human pathologies, such as cancer and aging [1–3]. Increasing evidence supports the idea that cell metabolism is intimately involved in the induction of adaptive response and the determination of cell fate decision in response to genotoxic stress [2, 4]. In turn, identification of the molecular pathways of metabolic stress response could provide important insight into the contribution of metabolism to DNA damage response and the development of strategies to treat or prevent cancer.

Glutamine (Gln), the most abundant amino acid in the body, provides a carbon source to fuel the mitochondrial tricaboxylic acid (TCA) cycle [5]. Gln metabolism has predominant roles in the decision of cells to commit to proliferate or undergo growth arrest. For example, highly proliferating cells exhibit an enhanced mitochondrial Gln metabolism, since they use precursors derived from the TCA cycle intermediates to synthesize lipids, proteins, and nucleic acids [6, 7]. Hence, it is not surprising that many cancer cells exhibit metabolic addiction to Gln [8]. Moreover, we demonstrated that mitochondrial Gln metabolism functions as an important regulator of cellular senescence [9]. Previously, it was shown that DNA damage represses mitochondrial Gln metabolism. Genotoxic stress suppresses the entry of Gln into the TCA cycle, which is required for proper DNA damage response, and a failure of this metabolic block induces impaired cell cycle arrest and delayed DNA repair [10]. Thus, these observations imply that mitochondrial Gln metabolism may have an important, previously undetermined role in cell survival upon DNA damage. However, it has not well investigated how mitochondrial Gln metabolism regulates DNA damage-induced cell death.

Amphiregulin (AREG), a member of the epidermal growth factor (EGF) family, is initially synthesized as a 252-amino acid precursor, pro-AREG, which is catalyzed by matrix metalloproteases at the plasma membrane and is then released as soluble AREG [11, 12]. AREG is frequently upregulated in many cancers, which is associated with poor prognosis [13]. Although it is well established that AREG acts as a ligand for EGF receptor by directly binding to it, AREG can translocate to the nucleus and affect global transcription [14, 15]. Interestingly, previous studies have found that AREG promotes apoptotic cell death in response to DNA damage. Upon DNA damage, tumor suppressor p53 induces AREG expression by binding to its promoter [16]. DNA damage-induced AREG is targeted to the nucleus and regulates precursor microRNA processing such as miR-15a, resulting in the repression of anti-apoptotic protein Bcl-2 [16].

Here, we describe a novel role of mitochondrial Gln metabolism in the regulation of cell death upon DNA damage by modulating AREG expression. Mechanistically, suppression of Gln metabolism increases the transcription of AREG by promoting the expression of Max-like protein X (MLX) transcription factor. We reveal that mitochondrial Gln metabolism determines the sensitivity of cancer cells after chemotherapy. Thus, our findings provide insights into how mitochondrial glutaminolysis affects cell survival in response to DNA damage and may open a new therapeutic research strategy to treat cancer.

## Results

### Mitochondrial glutamine (Gln) metabolism regulates cellular sensitivity to DNA damage

**Fig. 1:**
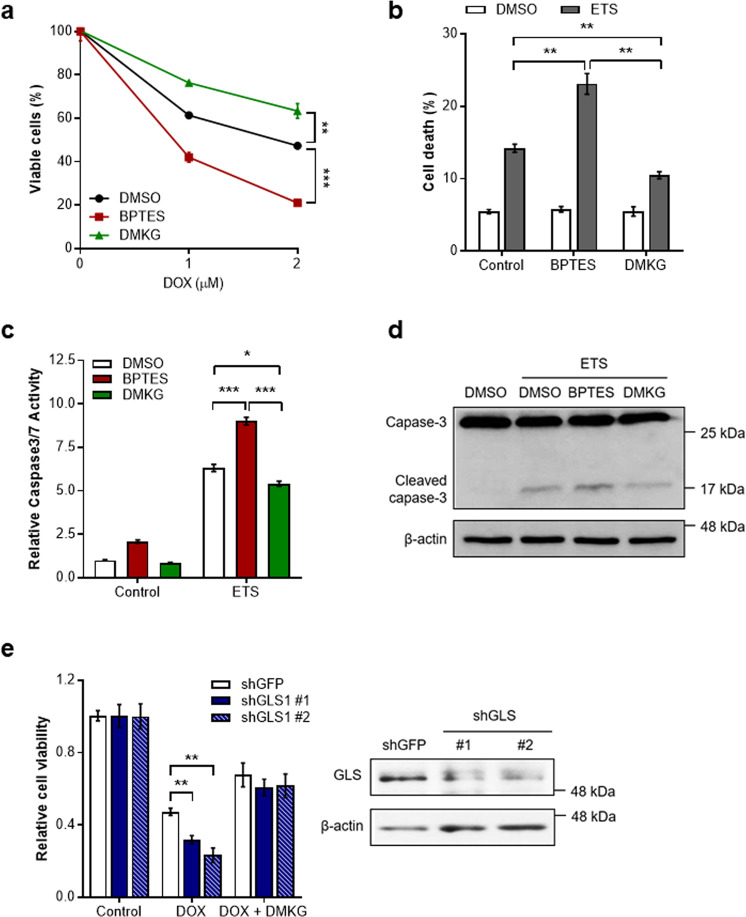
Mitochondrial Gln metabolism regulates DNA damage-induced cell death. **a** Cell viability of immortalized MEFs treated with DOX in the presence of BPTES or DMKG (*n* = 3). Statistical differences were determined using a two-way ANOVA. Data represent the mean ± SD. **b** Survival of cells treated with ETS in the presence of BPTES or DMKG (*n* = 3). Cell death was measured via propidium iodide exclusion assay. **c** Relative caspase3/7 activity of cells treated with ETS in the presence of BPTES or DMKG (*n* = 4). **d** Cleaved caspase 3 expression of cells treated with ETS in the presence of BPTES or DMKG. β-actin serves as a loading control. **e** Relative viability of cells expressing a control shRNA or two independent shRNAs to GLS1 incubated with or without DMKG upon DOX treatment (*n* = 3). Levels of GLS1 in cells expressing a control shRNA or shRNAs to GLS1 (right). All error bars ± SEM. *p < 0.05, **p < 0.01 and ***p < 0.001.

Since the repression of mitochondrial glutaminolysis has recently been linked to proper cellular DNA damage responses such as cell cycle arrest and DNA repair [10], we hypothesized that mitochondrial Gln metabolism could affect cell survival after DNA damage. Glutaminase (GLS) is essential for mitochondrial glutaminolysis, and thus GLS inhibition reduces the entry of Gln into the mitochondria. Interestingly, we found that treatment of bis-2-(5-phenylacetoamido-1,2,4-thiadiazol-2-yl)ethyl sulfide (BPTES), an inhibitor of GLS [17], markedly reduced cell viability in the presence of doxorubicin (DOX) [18], one of the most commonly used chemotherapeutic agents (Fig. 1a). In contrast, when we treated cell with dimethyl-αKG (DMKG) to increase mitochondrial glutaminolysis [19], DMKG made the cells more resistant to DOX treatment (Fig. 1a).

We next assessed whether mitochondrial Gln metabolism directly regulates DNA damage-induced cell death using DOX or another chemotherapeutic agent, etoposide (ETS) [20]. Consistent with previous results, Gln anaplerosis-inhibited cells exhibited significantly increased levels of cell death compared to control cells, whereas DMKG treatment led cells more resistant to DNA damage-induced cell death (Fig. 1b and Supplementary Fig. 1a). We also found that the increased cell death by GLS inhibition correlated with increased activities of caspase-3 and elevated levels of its cleaved form (Fig. 1c, d, and Supplementary Fig. 1b). Moreover, we obtained similar results in GLS knockdown cells (Fig. 1e and Supplementary Fig. 1c). Collectively, these data illustrate that mitochondrial Gln metabolism regulates apoptotic cell death in response to genotoxic stress.

### Mitochondrial Gln metabolism regulates AREG expression

**Fig. 2:**
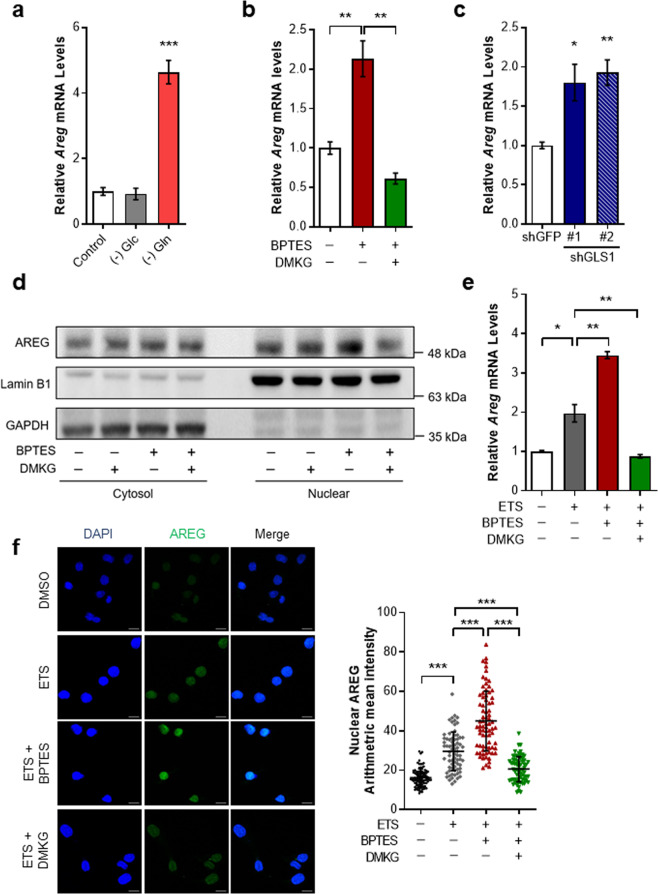
Mitochondrial Gln metabolism modulates AREG expression. **a** Relative AREG mRNA levels in immortalized MEFs incubated with or without Gln or Glc for 24 h (*n* = 3). β-actin was used as an endogenous control for qRT-PCR. **b** Relative AREG mRNA in cells treated with BPTES and/or DMKG (*n* = 3). **c** Relative AREG mRNA levels in HeLa cells expressing a control shRNA or two independent shRNAs to GLS (*n* = 3). **d** AREG protein levels in cytoplasmic and nuclear fractions of cells treated with BPTES, DMKG or both. GAPDH and LaminB1 were used as cytoplasmic or nuclear loading controls, respectively. **e** Relative AREG mRNA levels in cells treated with ETS in the presence of BPTES, DMKG or Both (*n* = 3). **f** Immunofluorescent staining was performed with nuclear marker (DAPI) and anti-AREG antibody on cells treated with ETS in the presence of BPTES or DMKG (DMSO: n = 100, ETS: *n* = 72, ETS + BPTES: *n* = 77, ETS + DMKG: *n* = 67). Scale bars represent 2 µm. The nuclear mean intensities of AREG as indicated (right). All error bars ± SEM. *p < 0.05, **p < 0.01 and ***p < 0.001.

As our results show that inhibition of Gln anaplerosis stimulates DNA damage-induced cell death, we sought to identify the molecular mechanism. Since a previous study has demonstrated that AREG induction upon DNA damage is required for apoptotic cell death [16], we hypothesized that inhibition of mitochondrial Gln metabolism augments DNA damage-induced cell death via AREG. To test this, we first examined whether Gln metabolism modulates AREG expression. In the absence of Gln, AREG messenger RNA (mRNA) levels were highly induced in immortalized mouse embryonic fibroblasts (MEFs) (Fig. 2a). However, we found no obvious increase of AREG expression under glucose-deprived conditions. Next, to confirm the role of mitochondrial glutaminolysis in AREG expression, we treated cells with multiple Gln anaplerosis inhibitors including BPTES, 6-diazo-5-oxo-L-norleucin (DON), and CB-839 [21] (Supplementary Fig. 2a). GLS inhibition markedly induced AREG expression (Fig. 2b and Supplementary Fig. 2b). Importantly, the induction of AREG upon Gln deprivation or GLS inhibition was almost completely restored after the addition of DMKG (Fig. 2b and Supplementary Fig. 2c). Likewise, GLS knockdown also significantly increased AREG (Fig. 2c). Gln can be further converted into αKG to replenish the TCA cycle either by glutamate dehydrogenase or transaminases. As further confirmation of the importance of mitochondrial Gln pathways on AREG expression, we treated cells with either aminooxyacetate (AOA) or epigallocatechin gallate (EGCG), selective inhibitors of transaminases and glutamate dehydrogenase, respectively (Supplementary Fig. 2a). Indeed, we observed a significant increase of AREG expression after either AOA or EGCG treatment (Supplementary Fig. 2d). These results confirm the significant role of mitochondrial Gln metabolism in AREG expression.

Because previous studies have proved that nuclear-localized AREG is implicated in the apoptotic response after DNA damage [16], we performed subcellular fractionation to examine whether GLS inhibition increases AREG expression in the nucleus. Importantly, AREG protein levels were significantly induced in the nuclear fractions of BPTES treated cells, and this increase was rescued by DMKG (Fig. 2d and Supplementary Fig. 2e). Next, to investigate the importance of AREG regulation by Gln anaplerosis in response to DNA damage, cells were treated with BPTES and/or DMKG followed by DNA damaging agent. As reported previously [16], AREG expression was induced after ETS treatment (Fig. 2e). Importantly, when combined with BPTES, this increase was notably augmented (Fig. 2e). In contrast, αKG supplement by DMKG was able to attenuate the induction of AREG upon DNA damage. Moreover, when we examined subcellular localization of AREG under this circumstance, near-identical results were observed in nucleus (Fig. 2f). In conclusion, these data demonstrate that mitochondrial Gln metabolism is an important modulator of AREG expression upon genotoxic stress.

### Mitochondrial Gln metabolism regulates the transcription of AREG through MLX

**Fig. 3:**
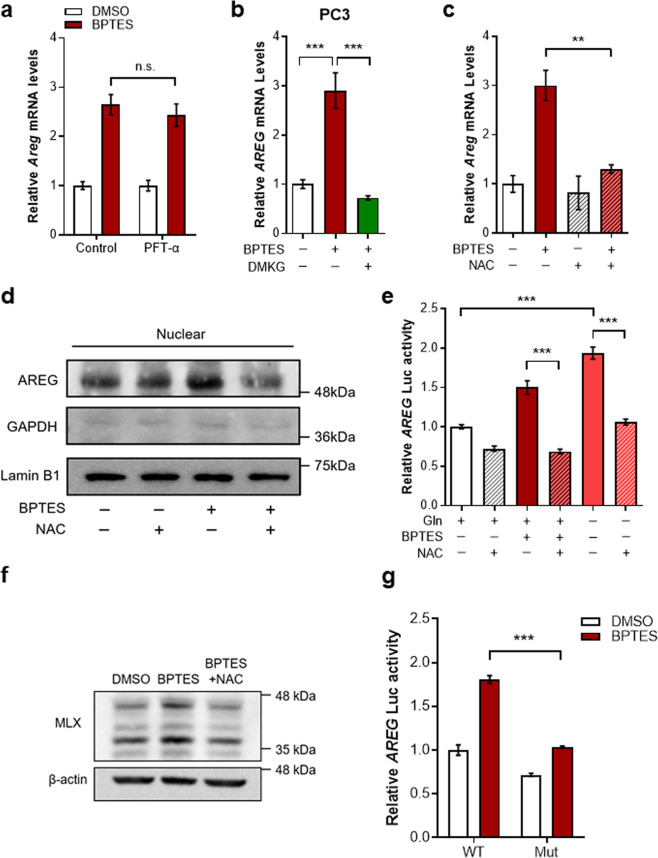
AREG is induced by MLX upon inhibition of Gln anaplerosis. **a** Relative AREG mRNA levels in immortalized MEFs treated with the indicated drugs (*n* = 3). **b** Relative AREG mRNA levels in PC3 cells treated with BPTES, DMKG or both (*n* = 3). **c** Relative AREG mRNA levels in cells treated with BPTES, NAC or both (*n* = 3). **d** AREG protein levels in nuclear fraction of cells treated with BPTES, NAC or both. LaminB1 was used as nuclear loading controls. **e** Luciferase activity was measured in cells treated with or without BPTES, Gln, and/or NAC as indicated (*n* = 3). **f** MLX protein levels in cells treated with or without BPTES, NAC or both. **g** Luciferase activity of WT or MLX-binding mutant AREG promoter in cells treated with or without BPTES (*n* = 3). All error bars ± SEM. n.s. not significant. **p < 0.01 and ***p < 0.001.

We next examined mechanisms underlying the regulation of AREG by mitochondrial Gln metabolism. Although it has been shown that tumor suppressor p53 binds to and modulates *AREG* promoter activity [16], the induction of AREG by GLS inhibition was not influenced in the presence of pifithrin-α, a p53 inhibitor (Fig. 3a). Consistent with these observations, we found that BPTES treatment elevated AREG expression in HEK293T cells containing inactive p53 and in p53-deficient PC3 human prostate cancer cells (Fig. 3b and Supplementary Fig. 3a), suggesting that p53 is unlikely to account for the observed AREG regulation by mitochondrial Gln metabolism.

Gln metabolism is intimately involved in maintaining cellular redox state. Gln is required for the synthesis of glutathione, a major antioxidant within cells, and Gln-derived aspartate supports redox homeostasis by creating NADPH [22]. Because mitochondrial Gln metabolism has essential roles in the cellular redox homeostasis, we speculated that the increased reactive oxygen species (ROS) levels by GLS inhibition may induce AREG expression. We first tested whether exogenous ROS exposure would increase AREG expression and found that AREG was induced by hydrogen peroxide treatment in a dose-dependent manner (Supplementary Fig. 3b). Next, cells were treated with the antioxidant N-acetylcysteine (NAC) to test whether suppressing ROS generation blocked the effects of GLS inhibition. Indeed, whereas AREG was induced in BPTES treated cells, NAC treatment dramatically rescued this induction (Fig. 3c, d and Supplementary Fig. 3c). Similar results were also observed with a cell-permeable glutathione analogue (Supplementary Fig. 3d). Because our data indicate that Gln anaplerosis-dependent regulation of AREG occurred at the mRNA level, we next examined the direct effects of GLS inhibition on AREG expression by measuring its promoter activity. In line with our findings, NAC treatment markedly blocked the induction of the *AREG* promoter activity upon BPTES treatment or Gln deprivation (Fig. 3e).

Next, to identify candidate transcription factors regulating AREG, we investigated the sequence of the human *AREG* promoter region. We used the TRANSFAC program, an algorithm available at http://genexplain.com, and identified 10 potential transcription factors that may bind and regulate *AREG* promoter activity (Supplementary Table 1). Interestingly, among these potential factors, MLX is involved in the control of metabolic processes including glucose metabolism [23]. Moreover, a sequence in the human *AREG* promoter exhibited significant homology with the canonical MLX recognition motif (Supplementary Fig. 3f). Thus, we inferred that MLX may regulate AREG induction by GLS inhibition. First, we found that BPTES treatment upregulated MLX protein levels, which was reversed by NAC treatment (Fig. 3f and Supplementary Fig. 3e), fitting with our model of ROS-dependent AREG induction as a result of GLS inhibition. Next, to determine whether MLX is directly involved in the Gln anaplerosis-dependent AREG regulation, we mutated a motif matching MLX consensus-binding site (Supplementary Fig. 3f) and measured the promoter activity after BPTES treatment. Indeed, mutation of the MLX binding site significantly abrogated the BPTES-induced increase in the activity of the *AREG* promoter (Fig. 3g). Together, our data suggest that enhanced ROS production by suppression of Gln anplerosis contributes to enhanced AREG transcription via MLX.

### Mitochondrial Gln metabolism regulates DNA damage-induced cell death in an AREG-dependent fashion

**Fig. 4:**
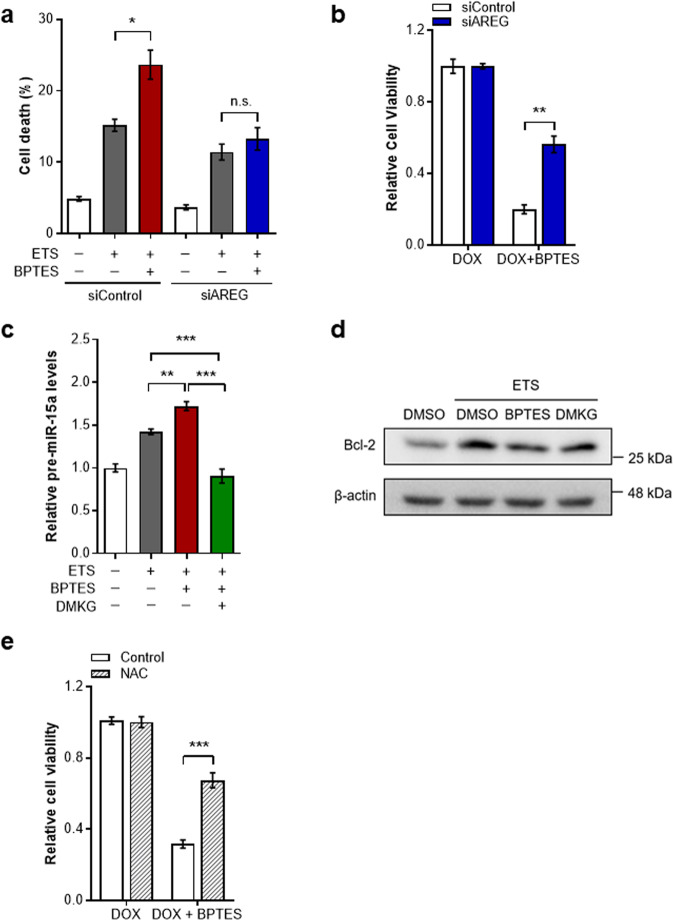
Mitochondrial Gln metabolism regulates DNA damage-induced cell death through AREG. **a** Cell death of immortalized MEFs transfected with nontargeting siRNA (siControl) or with siRNA against AREG (siAREG). Cells were treated with or without ETS, BPTES, or both (*n* = 3). **b** Cell viability of siControl or siAREG cells treated with or without DOX, BPTES or both (*n* = 3). **c** Cells were treated with the indicated drugs (*n* = 5). Total RNA was amplified with pre-miR-15a specific primers. **d** Bcl-2 protein levels in cells treated with ETS in the presence of BPTES or DMKG. **e** Cell viability of control or NAC-treated cells incubated with or without DOX, BPTES, or both (*n* = 4). All error bars ± SEM. n.s., not significant. *p < 0.05, **p < 0.01 and ***p < 0.001.

Our results show that mitochondrial Gln metabolism modulates AREG expression after DNA damage, which prompted us to investigate whether Gln anaplerosis potentially controls DNA damage-induced cell death via regulation of AREG. We suppressed AREG expression by using short interfering RNA and assessed the effect of Gln anaplerosis for cell survival followed by ETS treatment. As expected, BPTES treatment led cells to be more vulnerable to DNA damage-induced cell death (Fig. 4a). However, GLS inhibition had no further effect on cell death in AREG knockdown cells, underscoring the role of AREG in Gln anaplerosis-mediated regulation of cell death (Fig. 4a and Supplementary Fig. 4a). Additionally, the markedly decreased cell viability in the presence of BPTES was significantly abrogated in AREG knockdown cells (Fig. 4b and Supplementary Fig. 4b, 4c and 4d).

Given that AREG, induced by DNA damage, promotes microRNA processing such as miR-15a to reduce the expression of anti-apoptotic protein Bcl-2 [16], we first examined whether Gln anaplerosis modulates pre-miR-15a levels in response to DNA damage. Whereas the DNA damage-triggered increase of pre-miR-15a was significantly higher in Gln anaplerosis-inhibited cells, this induction was significantly reduced by DMKG treatment (Fig. 4c). Because the reduction of Bcl-2 by AREG contributes to the increased cell death after DNA damage [16], we next assessed protein levels of Bcl-2 under these conditions. In line with this model, Bcl-2 expression was decreased by BPTES, which was rescued by DMKG treatment (Fig. 4d and Supplementary Fig. 4e). As we observed that increased ROS levels by GLS inhibition are responsible for AREG induction, we tested whether suppressing ROS could affect the decreased cell survival after GLS inhibition. Consistent with the reversion of AREG expression by NAC (Fig. 3c), NAC treatment significantly diminished the additive effect of BPTES treatment on cell viability after DNA damage (Fig. 4e and Supplementary Fig. 4f). Together, our data demonstrate that mitochondrial Gln metabolism regulates DNA damage-induced cell death through AREG.

### Mitochondrial Gln metabolism determines drug sensitivity of cancer cells after chemotherapy

**Fig. 5:**
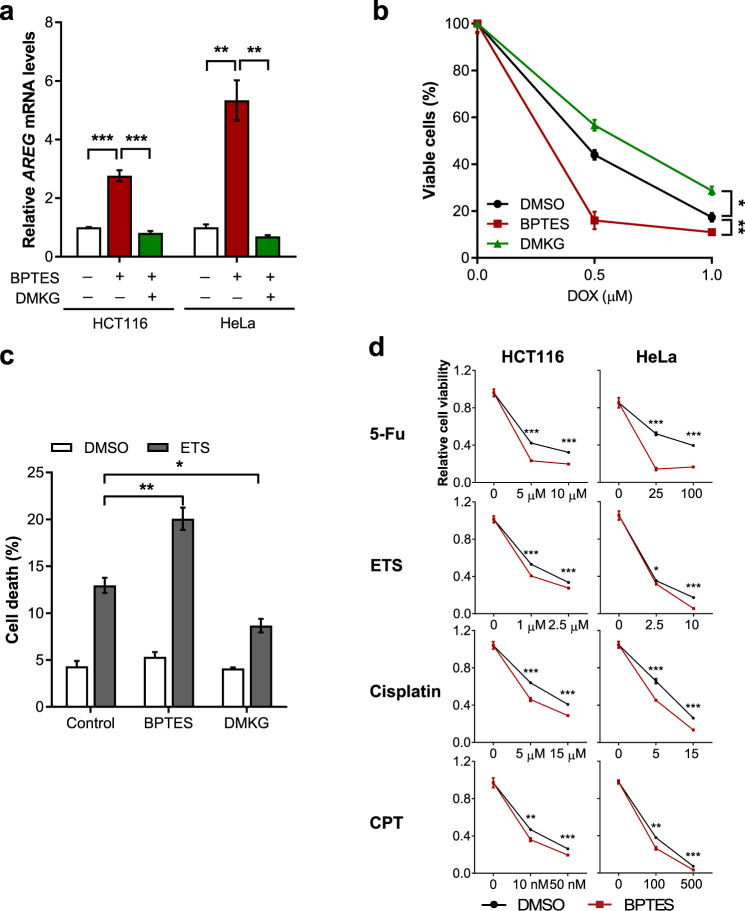
Inhibition of Gln anaplerosis sensitizes cancer cells to chemotherapy. **a** Relative AREG mRNA levels in HCT116 and HeLa cells treated as indicated (*n* = 3). **b** Cell viability of HeLa cells treated with DOX in the presence of BPTES or DMKG (*n* = 3). Statistical differences were determined using a two-way ANOVA. Data represent the mean ± SD. **c** Cell death of HeLa cells treated with or without ETS in the presence of BPTES or DMKG (*n* = 3). **d** Cell viability of HCT116 or HeLa cells treated with the indicated chemotherapeutic agents in the presence of DMSO or BPTES (*n* = 3). All error bars ± SEM. *p < 0.05, **p < 0.01 and ***p < 0.001.

Chemotherapy, designed to kill cancer cells by inducing DNA damage, is one of the most common cancer therapies for treating various types of cancers [24]. However, some cancer cells exhibit profound resistance to DNA damage-induced cell death, which is a major limitation for DNA damaging therapy applications [25]. Our data indicate that inhibition of Gln anaplerosis promotes the final death cell fate after DNA damage. These observations suggest that targeting mitochondrial Gln anaplerosis offers an interesting therapeutic approach for cancer cells. To test this concept, we examined whether inhibition of Gln metabolism would increase cell death by chemotherapeutic agents. We found that GLS inhibition or Gln deprivation-induced AREG expression in cancer cell lines including HeLa and HCT116, which was reversed by DMKG treatment (Fig. 5a and Supplementary Fig. 5a). Consistent with our prediction, the inhibition of Gln anaplerosis by BPTES rendered both cancer cells more susceptible to DOX treatment (Fig. 5b and Supplementary Fig. 5b) and produced notably augmented DNA damage-induced cell death (Fig. 5c and Supplementary Fig. 5c).

To further assess the broader relevance of this pathway in cancer cell survival after chemotherapy, we treated cells with various chemotherapeutic agents. HeLa or HCT116 cells were co-treated with BPTES during the drug pulse and assessed cell viability. Importantly, the combinational treatment produced synergistic effects in both cell lines (Fig. 5d). Thus, our results demonstrate that the treatment aiming at targeting Gln anaplerosis augments cell death by DNA damaging agents.

### Inhibition of mitochondrial Gln metabolism synergizes with chemotherapeutic agents to induce cell death in tumors

**Fig. 6:**
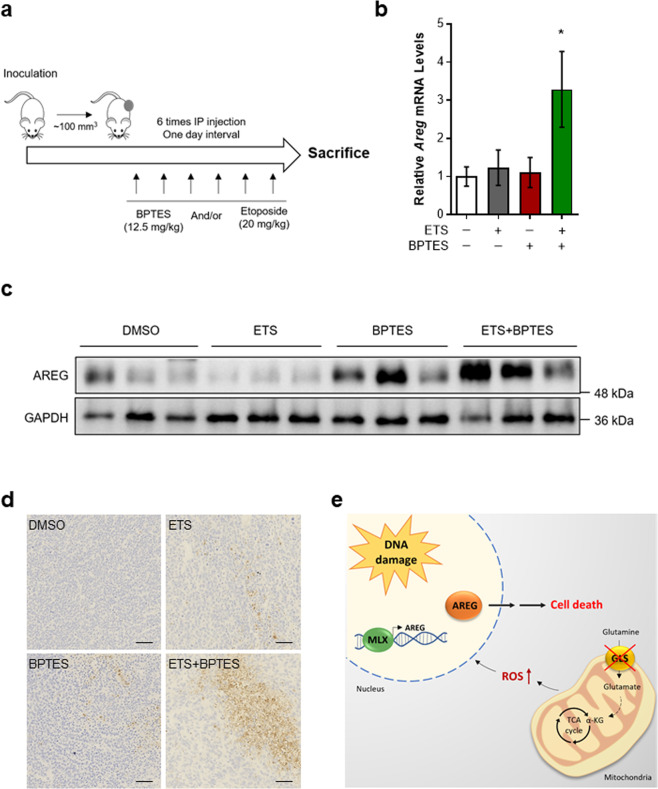
The combination of GLS inhibition with chemotherapeutic agents is effective to induce cell death in tumors. **a** Animal experiment schedule. Mice were injected subcutaneously with transformed MEFs. Drugs were administered as indicated. A day after the treatment, mice were sacrificed and tumors were collected. **b, c** Relative AREG mRNA (**b**) and protein (**c**) levels in allograft tumors from mice treated with or without ETS, BPTES, or both (*n* = 3). **d** Immunohistochemical analysis of cleaved caspase-3 expression in allograft tumors. Scale bars represent 50 µm. **e** A proposed model illustrating the regulation of DNA damage-induced cell death by mitochondrial Gln metabolism. All error bars ± SEM. *p < 0.05.

In order to expand our results to in vivo, we performed allograft assays with E1A and Ras transformed MEFs. The mice were treated with ETS, BPTES or both, when tumors reached a volume of approximately 100 mm^3^ (Fig. 6a). We used the selected doses of BPTES and ETS that had minimal effects as a single treatment in mice. First, we examined whether GLS inhibition augments AREG expression after DNA damage. Accordant with our cellular models, tumors from mice injected with BPTES in combination with ETS exhibited enhanced AREG mRNA levels (Fig. 6b). Moreover, this combination demonstrated higher levels of AREG protein than ETS treatment alone (Fig. 6c).

Next, we tested whether Gln inhibition determines the sensitivity of tumors after chemotherapy by performing immunohistochemistry. Whereas single-agent treatments only mildly affected levels of activated caspase-3 at these concentrations, the combination of BPTES and ETS resulted in enhanced number of cleaved caspase-3 positive cells (Fig. 6d and Supplementary Fig. 5d). Taken together, our results demonstrate that tumors are noticeably more vulnerable to chemotherapy when mitochondrial Gln metabolism is inhibited.

## Discussion

In this study, we demonstrate that mitochondrial Gln metabolism regulates DNA damage-induced cell death through AREG (Fig. 6e). Previously, it has been reported that induction of AREG is required for the apoptotic response to DNA damage [16]. Our study shows that elevated ROS by GLS inhibition additionally contributes to increased transcription of AREG after DNA damage. Significantly, AREG knockdown attenuates the increase of DNA damage-induced cell death by GLS inhibition. This idea is further validated by the finding that modulation of Gln anaplerosis determines sensitivity of cancer cells to chemotherapeutic drugs, highlighting the potential importance of this pathway in cancer therapy. Our studies, therefore, uncover the role of mitochondrial Gln metabolism in determining the survival-death cell fate decision in response to DNA damage with important implications for cancer treatment.

Our findings are consistent with previous work showing that the repression of mitochondrial Gln metabolism is required for proper cellular DNA damage response [10]. Jeong et al. (2013) found that DNA damage triggers a block in the entry of Gln into the TCA cycle, which is required for the DNA damage responses such as cell cycle arrest and DNA repair, and defects of this lead to DNA damage-induced genomic instability, resulting in tumorigenic phenotypes. In this study, we demonstrate that pharmacological interference in Gln anaplerosis unequivocally resulted in increased cell death after DNA damage. Because an inadequate elimination of DNA damaged cells often leads to the accumulation of chromosomal instability and tumor development, our study and the one by Jeong et al. reveal that the repression of mitochondrial Gln metabolism in response to genotoxic stress is an important cellular metabolic checkpoint for sustaining genomic stability and preventing tumorigenesis.

Although our work shows that the regulation of AREG by Gln anaplerosis is critical for DNA damage-induced cell death, it does not exclude the involvement of other Gln-dependent pathways that may contribute to cell survival upon DNA damage. For example, Gln is important for cellular redox balance by supporting de novo synthesis of glutathione [26], an antioxidant that functions as a critical regulator in cellular defense against oxidative DNA damage. Gln metabolism also supports the synthesis of nucleotides and NADPH [22], which are essential for the repair of damaged DNA. Additionally, liver-type cells also express GLS2 [27], which is induced by DNA damage via p53-dependent manner [28]. Thus, it will be interesting for future work to examine whether other branches of Gln metabolism affect cell survival after genotoxic stress.

On the other hand, cancer cells exhibit altered metabolic features, including increased glycolysis and decreased oxidative phosphorylation [29]. Cancer cells are more hypoxic than normal cells, as determined by increased hypoxia-inducible factor-1α (HIF1α) protein levels. As HIF1α inhibits pyruvate dehydrogenase (PDH) which converts pyruvate to acetyl-CoA, Gln is essential for the maintenance of the integrity of TCA cycle in cancer cells by refilling the mitochondrial carbon pool [8]. Moreover, GLS inhibition can activate HIF1α by decreasing αKG, which may lead to further PDH inhibition. Therefore, it is possible that the inhibition of Gln anaplerosis leads to a decrease in TCA cycle intermediates and then makes cancer cells more sensitive to DNA damage. This idea is also supported by the fact that NAC can improve the TCA cycle intermediates by stimulating carbon flux through pyruvate decarboxylase [30]. Furthermore, some cancer cells are capable of recovering Gln anaplerosis upon GLS inhibition by activating alternative metabolic pathways [31, 32]. Therefore, we speculate that cancer cells, which could exploit adaptative pathways upon the repression of Gln anaplerosis by DNA damage, may also exhibit resistance to DNA damaging agents.

Although we have focused on the elimination of cancer cells by inhibiting Gln anaplerosis during chemotherapy, there are positive aspects of Gln metabolism for cancer patients. For example, intestinal epithelial tissues and immune systems require energy to fight cancers and to recover from genotoxic stresses induced by chemotherapy or radiation therapy [29]. Moreover, Gln consumption is required to restore reduced glutathione after DNA damage and promote the regeneration process of these tissues [29]. Thus, further work will be needed to determine the exact roles of Gln metabolism during chemotherapy.

Our studies shed light on potential therapeutic strategies for cancer treatment. DNA damaging therapies, such as chemotherapy and radiotherapy, are widely used as the standard care for cancer treatment [3, 33]. However, because many cancer cells retain the capacity to develop resistance to DNA damage by activating repair machineries and/or anti-apoptotic pathways, achieving a successful therapeutic index remains a major obstacle to the development of effective cancer therapy [25, 34]. Importantly, we demonstrate that the induction of AREG by GLS inhibition sensitizes cancer cells to DNA damage. Therefore, we speculate that targeting this molecular pathway in cancer cells can potentially synergize with therapies that induce DNA damage. Given that most cancer cells are dependent on Gln metabolism for their growth [8, 35] and clinical grade GLS inhibitors are being developed to target this reliance on cancer cells [36], our findings may have more significant therapeutic implications.

In sum, we identified a causal link between mitochondrial Gln metabolism and DNA damage-induced cell death involving AREG. Moreover, our studies underscore the significance of metabolic stress response in cell fate decision under genotoxic stress and, importantly, support the development of therapeutic strategies targeting mitochondrial Gln metabolism as cancer treatment.

## Materials and methods

### Cell culture

MEFs were immortalized using the SV40 large T antigen or were transformed with E1A and Ras at passage 4. MEFs, HEK293T, HeLa and HCT116 cell lines used in this work have been described before [9, 32]. Cells were maintained in Dulbecco’s modified Eagle’s medium (Welgene, Gyeongsan, Republic of Korea) supplemented with 10% fetal bovine serum (Gibco, NY, USA) and penicillin/streptomycin (Biowest, Nuaillé, France). All cell lines were tested routinely for mycoplasma contamination by using a commercially available kit (Takara Bio Inc, Shinga, Japan).

### Constructs and reagents

The following antibodies were used: GLS (#ab93434, Abcam, Cambridge, MA, USA), AREG (#sc74501, Santa Cruz Biotechnology, Dallas, USA), MLX (#85570 s, Cell signaling, Danvers, MA, USA), Lamin B1 (#12586 s, Cell signaling), caspase 3 (#9664 s, Cell signaling), GAPDH (#CSB-PA00025A0Rb, Cusabio biotech, Wuhan, China), Bcl-2 (#CSB-PA000983, Cusabio biotech) and β-actin (#ABT264, Sigma, St. Louis, MO, USA). AOA, EGCG, DMKG, NAC, glutathione, DOX, cisplatin, camptothecin, ETS, and 5-fluorouracil were purchased from Sigma. BPTES was purchased from Cayman Chemical (Ann Arbor, MI, USA). CB-839 was obtained from Selleckchem (Houston, TX, USA). shRNAs for AREG (SHCLNDTRCN0000117992 and SHCLNDTRCN0000117996) were purchased from Sigma. shRNAs for GLS were described previously [10].

### Western blot analysis

Cells were lysed with EZ-RIPA lysis buffer (ATTO, Tokyo, Japan) supplemented with a protease inhibitor (ATTO) and a phosphatase inhibitor (ATTO). Cell lysates were separated by sodium dodecyl sulfate polyacrylamide gel electrophoresis (SDS-PAGE). After SDS-PAGE, proteins were transferred to Nitrocellulose membrane. Membranes were blocked for 1 h in Tris-buffered saline (TBS) containing 5% Bovine Serum Albumin (BSA) and 0.1% Tween 20 (TBS-T) and subsequently were incubated with the primary antibody overnight at 4 °C. The membranes were washed with TBS-T and incubated with a horseradish peroxidase-conjugated secondary antibody for 1 h. Blots were visualized by ECL (Promega, Madison, WI, USA) and Luminescent Image Analysis System.

### RNA analysis

Total RNA was prepared with RNAiso Plus (Takara Bio Inc, Shinga, Japan) according to the manufacturer’s instructions. Briefly, 0.5 μg of total RNA was reverse-transcribed using the iScript cDNA synthesis kit (Takara Bio Inc). Diluted cDNAs were analyzed by real-time PCR using SYBR Green I Mastermix on a Lightcycler 480 (Roche, South San Francisco, CA, USA). The level of gene expression was normalized to β-actin. Primers sequences were: GCTGTCGCTCTTGATACTCG and CTTCCCAGAGTAGGTGTCATTG for human *AREG*; CTACGTCGCCCTGGACTTCGAGC and GATGGAGCCGCCGATCCACACGG for human *β-Actin*; CACCATAAGCGAAATGCCTTC and TCTTGGGCTTAATCACCTGTTC for mouse *Areg*; AGCCATGTACGTAGCCATCC and CTCTCAGCTGTGGTGGTGAA for mouse *β-Actin*. For the detection of precursor form of miR-15a (pre-miR-15a), cDNAs were synthesized using ReverTra Ace™ qPCR RT kit (Toyobo, Osaka, Japan). The relative levels of pre-miR-15a were determined by RT-qPCR using a specific primer set (Fwd: 5ʹ-CCCTTGGAGTAAAGTAG-3ʹ and Rev: 5ʹ-TCCTTGTATTTTGAGGC-3ʹ). GAPDH mRNA was used for the normalization.

### siRNA Transfections

Twenty-five (25) nM siRNAs were transfected in cells right after being seeded at a density of 30–50% confluency depending on experiments using Lipofectamine RNAiMAX (Invitrogen, Carlsbad, CA, USA) according to the manufacturer’s protocols. Cells were harvested 24–36 h post-transfection as described in the figure legends. siRNAs for AREG were purchased from Sigma (cat#: EMU025631).

### Flow cytometric measurement

Cells at less than 80% confluence were treated with DNA damage agents. After treatment, cells were harvested by trypsinization, pelleted by centrifugation, and resuspended in PBS containing 3% fetal bovine serum. The measurement of cell death was performed by flow cytometry using propidium iodide (PI) staining, as previously described.

### Cell viability assay

Cells were plated into 96-well plates at 1000 cells per well in 100 μl of growth media. The following day, growth media was replaced with that containing ETS or DOX. Parallel plates were analyzed at 3 days by Cell Titer Glo analysis (Promega), per the manufacturer’s instruction.

### Immunofluorescence assay

Cells were fixed with 4% paraformaldehyde for 5 min at room temperature and permeablized for 20 min on 0.5% PBST (PBS containing 0.5% Triton X-100). After PBS washing twice, the permeablized cells were blocked on 5% normal goat serum in 0.1% PBST (PBS containing 0.1% Triton X- 100) for 1 h and then the cells were incubated with AREG antibody (R&D systems, Minneapolis, MN, USA) in 0.1% PBST with 5% NGS overnight at 4 °C. After washing three times in 0.1% PBST, cells were stained with FITC-conjugated streptavidin antibody for 1 h at room temperature. Finally, cells were washed five times in 0.1% PBST and mounted with Vectashield mounting medium with DAPI (Vector Laboratories, Burlingame, CA, USA). The fluorescence signal was detected using confocal microscopy.

### Immunohistochemistry

Tumors were fixed in 4% paraformaldehyde, embedded in paraffin, and sectioned at 3 μM thickness. Paraffin-embedded tumor sections were deparaffinized, rehydrated, and incubated with citrate buffer (ImmunoBioScience, Mukilteo, WA, USA) 95 °C for antigen retrieval. To quench endogenous peroxidase activity, the slides were incubated in 1.4% hydrogen peroxide/methanol. After blocking with 2.5% normal horse serum (Vector Laboratories) for 1 h, the slides were incubated with anti-cleaved Caspase3 antibody (Cell signaling) at 4 °C overnight. After PBS washing three times, the slides were incubated with a micropolymer HRP-conjugated secondary antibody (Vector Laboratories) for 1 h at room temperature. Antigens were demonstrated by DAB (Vector Laboratories). The slides were counterstained with EASYSTAIN Harris Hematocylin (YD diagnostics, Yongin, Korea). Finally, the slides were dehydrated in graded ethanol and mounted with Permount mounting medium (Fisher Scientific, Waltham, MA, USA). The images were acquired with Axio Scan.Z1 (ZEISS, Oberkochen, Germany).

### Animal studies

All animal experiments were undertaken in accordance with the National Institutes of Health’s Guide for the Care and Use of Laboratory Animals, with approval of the Animal Experiment Ethics Committee of the Catholic University of Korea College of Medicine. A total of 20 male BALB/c nude mice were purchased from Orient Bio (Seongnam, Korea). Eight-week-old BALB/c nude mice were randomly divided into four groups with five mice in each group and were injected subcutaneously on the abdominal flank with 1 × 10^6^ transformed MEFs in 50 μl PBS mixed with 50 μl Matrigel. Mice were divided into four groups (control, BPTES (12.5 mg/kg), ETS (20 mg/kg), BPTES plus ETS). When the tumor volumes reached approximately 100 mm^3^, BPTES and/or ETS were intraperitoneally administered 6 times in total at a one-day interval. A day after a course of treatment, mice were sacrificed for tumor tissue collection.

### Reporter assay

The human AREG promoter was cloned into pGL3 basic vector to obtain a pGL3-AREG promoter plasmid. pGL3-AREG promoter was co-transfect with Renilla luciferase-expressing pRL-TK. After 18 h of transfection, the cells were treated with drugs for 24 h. Luciferase activity was then assayed according to the manufacturer’s instructions (Promega). Firefly luciferase activity was normalized to Renilla luciferase activity. Mutations in the AREG promoter were generated with the Quickchange kit (Agilent Technologies, CA, USA) and primer sequences were: GCGAATCCTTACGAAAAAGGGAGGCGGGGCG and CGCCCCGCCTCCCTTTTTCGTAAGGATTCGC.

### Statistical analysis

Unpaired two-tailed Student’s *t* test was performed unless otherwise noted. All experiments were performed at least two or three independent experiments and the number of each sample was more than 3 (*n* ≥ 3).

## Supplementary information

supplementary information

## Data Availability

All data generated or analysed during this study are included in this published article and its supplementary information files.

## References

[1] Ciccia A, Elledge SJ (2010). The DNA damage response: making it safe to play with knives. Mol. Cell.

[2] Turgeon MO, Perry NJS, Poulogiannis G (2018). DNA damage, repair, and cancer metabolism. Front Oncol.

[3] Lord CJ, Ashworth A (2012). The DNA damage response and cancer therapy. Nature.

[4] Zhu JJ, Thompson CB (2019). Metabolic regulation of cell growth and proliferation. Nat. Rev. Mol. Cell Bio.

[5] Jones RG, Thompson CB (2009). Tumor suppressors and cell metabolism: a recipe for cancer growth. Genes Dev.

[6] Altman BJ, Stine ZE, Dang CV (2016). From Krebs to clinic: glutamine metabolism to cancer therapy. Nat. Rev. Cancer.

[7] Cluntun AA, Lukey MJ, Cerione RA, Locasale JW (2017). Glutamine metabolism in cancer: understanding the heterogeneity. Trends Cancer.

[8] Wise DR, Thompson CB (2010). Glutamine addiction: a new therapeutic target in cancer. Trends Biochem Sci.

[9] Yang S, Hwang S, Kim M, Seo SB, Lee JH, Jeong SM (2018). Mitochondrial glutamine metabolism via GOT2 supports pancreatic cancer growth through senescence inhibition. Cell Death Dis.

[10] Jeong SM, Xiao C, Finley LW, Lahusen T, Souza AL, Pierce K (2013). SIRT4 has tumor-suppressive activity and regulates the cellular metabolic response to DNA damage by inhibiting mitochondrial glutamine metabolism. Cancer Cell.

[11] Brown CL, Meise KS, Plowman GD, Coffey RJ, Dempsey PJ (1998). Cell surface ectodomain cleavage of human amphiregulin precursor is sensitive to a metalloprotease inhibitor. Release of a predominant N-glycosylated 43-kDa soluble form. J. Biol. Chem.

[12] Berasain C, Avila MA (2014). Amphiregulin. Semin Cell Dev. Biol.

[13] Busser B, Sancey L, Brambilla E, Coll JL, Hurbin A (2011). The multiple roles of amphiregulin in human cancer. Biochim Biophys. Acta.

[14] Isokane M, Hieda M, Hirakawa S, Shudou M, Nakashiro K, Hashimoto K (2008). Plasma-membrane-anchored growth factor pro-amphiregulin binds A-type lamin and regulates global transcription. J. Cell Sci.

[15] Johnson GR, Saeki T, Auersperg N, Gordon AW, Shoyab M, Salomon DS (1991). Response to and expression of amphiregulin by ovarian carcinoma and normal ovarian surface epithelial cells: nuclear localization of endogenous amphiregulin. Biochem Biophys. Res Commun.

[16] Taira N, Yamaguchi T, Kimura J, Lu ZG, Fukuda S, Higashiyama S (2014). Induction of amphiregulin by p53 promotes apoptosis via control of microRNA biogenesis in response to DNA damage. Proc. Natl Acad. Sci. USA.

[17] Robinson MM, McBryant SJ, Tsukamoto T, Rojas C, Ferraris DV, Hamilton SK (2007). Novel mechanism of inhibition of rat kidney-type glutaminase by bis-2-(5-phenylacetamido-1,2,4-thiadiazol-2-yl)ethyl sulfide (BPTES). Biochem J.

[18] Yang F, Teves SS, Kemp CJ, Henikoff S (2014). Doxorubicin, DNA torsion, and chromatin dynamics. Biochim Biophys. Acta.

[19] Jeong SM, Hwang S, Seong RH (2016). SIRT4 regulates cancer cell survival and growth after stress. Biochem Biophys. Res Commun.

[20] Montecucco A, Zanetta F, Biamonti G (2015). Molecular mechanisms of etoposide. EXCLI J.

[21] Gross MI, Demo SD, Dennison JB, Chen L, Chernov-Rogan T, Goyal B (2014). Antitumor activity of the glutaminase inhibitor CB-839 in triple-negative breast cancer. Mol. Cancer Ther.

[22] Yoo HC, Yu YC, Sung Y, Han JM (2020). Glutamine reliance in cell metabolism. Exp. Mol. Med.

[23] Havula E, Hietakangas V (2012). Glucose sensing by ChREBP/MondoA-Mlx transcription factors. Semin Cell Dev Biol.

[24] Schirrmacher V (2019). From chemotherapy to biological therapy: a review of novel concepts to reduce the side effects of systemic cancer treatment (Review). Int J Oncol.

[25] Mansoori B, Mohammadi A, Davudian S, Shirjang S, Baradaran B (2017). The different mechanisms of cancer drug resistance: a brief review. Adv Pharm Bull.

[26] Alberghina L, Gaglio D (2014). Redox control of glutamine utilization in cancer. Cell Death Dis.

[27] Curthoys NP, Watford M (1995). Regulation of glutaminase activity and glutamine metabolism. Annu Rev Nutr.

[28] Suzuki S, Tanaka T, Poyurovsky MV, Nagano H, Mayama T, Ohkubo S (2010). Phosphate-activated glutaminase (GLS2), a p53-inducible regulator of glutamine metabolism and reactive oxygen species. Proc. Natl Acad. Sci. USA.

[29] Michalak KP, Mackowska-Kedziora A, Sobolewski B, Wozniak P (2015). Key roles of glutamine pathways in reprogramming the cancer metabolism. Oxid Med Cell Longev.

[30] Zwingmann C, Bilodeau M (2006). Metabolic insights into the hepatoprotective role of N-acetylcysteine in mouse liver. Hepatology.

[31] Biancur DE, Paulo JA, Malachowska B, Quiles Del Rey M, Sousa CM, Wang X (2017). Compensatory metabolic networks in pancreatic cancers upon perturbation of glutamine metabolism. Nat Commun.

[32] Kim M, Gwak J, Hwang S, Yang S, Jeong SM (2019). Mitochondrial GPT2 plays a pivotal role in metabolic adaptation to the perturbation of mitochondrial glutamine metabolism. Oncogene.

[33] Hosoya N, Miyagawa K (2014). Targeting DNA damage response in cancer therapy. Cancer Sci.

[34] Vasan N, Baselga J, Hyman DM (2019). A view on drug resistance in cancer. Nature.

[35] Zhang J, Pavlova NN, Thompson CB (2017). Cancer cell metabolism: the essential role of the nonessential amino acid, glutamine. EMBO J.

[36] Song M, Kim SH, Im CY, Hwang HJ (2018). Recent development of small molecule glutaminase inhibitors. Curr Top Med Chem.

