# Supplementary material for: Mitochondrial glutamine metabolism regulates sensitivity of cancer cells after chemotherapy via amphiregulin
Source: Cell Death Discov. 2021 Dec 20;7:395. doi: 10.1038/s41420-021-00792-7 (PMC8685276; doi:10.1038/s41420-021-00792-7)

**Supplementary figure legends**

**Supplementary figure 1: Mitochondrial Gln metabolism regulated DNA damage-induced cell death**

**a** Survival of cells treated with DOX in the presence of BPTES or DMKG. Cell death was measured via propidium iodide exclusion assay. **b** Cleaved caspase 3 expression in whole-cell lysates from cells treated with ETS in the presence of BPTES or DMKG. **c** Survival of cells expressing a control shRNA or two independent shRNAs to GLS1 incubated with or without DMKG upon DOX treatment. Cell death was measured via propidium iodide exclusion assay. All error bars ±SEM. *p < 0.05, **p < 0.01 and ***p < 0.001.

**Supplementary figure 2: Mitochondrial Gln metabolism regulates AREG expression.**

**a** A diagram showing the enzymes involved in Gln anaplerosis and the inhibitors used in this study. **b** Relative AREG mRNA levels in immortalized MEFs treated with or without DON or CB-839. **c** Relative AREG mRNA levels in cells cultured in standard media or media without Gln in the presence of DMSO or DMKG. **d** Relative AREG mRNA levels in cells treated with or without EGCG or AOA. **e** Nuclear AREG protein levels of cells treated with BPTES, DMKG or both. All error bars ±SEM. *p < 0.05, **p < 0.01 and ***p < 0.001.

**Supplementary figure 3: Mitochondrial Gln metabolism regulates AREG via ROS.**

**a** Relative AREG mRNA levels in HEK293T cells treated with or without BPTES or BPTES + DMKG. **b** Relative AREG mRNA levels in immortalized MEFs treated with the indicated doses of hydrogen peroxide (H_2_O_2_). **c** AREG protein levels in nuclear fraction of cells treated with BPTES, NAC or both. MLX protein levels in cells treated with or without BPTES, NAC or both. **d** Relative AREG mRNA levels in cells treated with or without BPTES, GSH or both. **e** MLX protein levels in cells treated with or without BPTES, NAC or both. **f** MLX-binding consensus motif on the human AREG gene promoter. Sequences of WT and MLX-binding site mutated (Mut) AREG promoters. All error bars ±SEM. *p < 0.05 and **p < 0.01.

**Supplementary figure 4: AREG is responsible for the Gln anaplerosis-mediated cell death upon DNA damage.**

**a** Relative AREG mRNA levels in immortalized MEFs transfected with nontargeting siRNA (siControl) or with siRNA against AREG (siAREG). **b** Cell viability of siControl or siAREG cells treated with or without DOX, BPTES or both. **c** Relative AREG mRNA levels in MEFs expressing control shRNA (shGFP) or two shRNAs targeted against AREG (shAREG). **d** Cell viability of shGFP or shAREG cells treated with or without DOX, BPTES or both. **e** Bcl-2 protein levels in cells treated with ETS in the presence of BPTES or DMKG. **f** Cell viability of control or NAC-treated cells incubated with or without DOX, BPTES or both. All error bars ±SEM. n.s., not significant. *p < 0.05, **p < 0.01 and ***p < 0.001.

**Supplementary figure 5: The pharmacologic inhibition of Gln anaplerosis synergizes with chemotherapeutic agents.**

**a** Relative AREG mRNA levels in HCT116 and HeLa cells cultured in standard media or media without Gln in the presence of DMKG. **b** Cell viability of HCT116 cells treated with DOX in the presence of BPTES or DMKG. **c** Cell death of HCT116 cells treated with or without ETS in the presence of BPTES or DMKG. **d** The mean intensities of cleaved caspase-3 from tumor tissues treated with or without ETS, BPTES or both. All error bars ±SEM. *p < 0.05, **p < 0.01 and ***p < 0.001.

**Supplementary Table 1: Putative transcription factor-recognition sites on the human *AREG* promoter**

| **Gene Symbol** | **Gene Name** |  |
| --- | --- | --- |
| **KLF4** | **Krueppel-like factor 4** | |
| **KLF6** | **Krueppel-like factor 6** | |
| **ATF2** | **Activating transcription factor 2** | |
| **Ets** | **E26 transformation-specific** | |
| **AP-2** | **Activator protein-2** | |
| **MLX** | **Max-like protein X** | |
| **CREB** | **cAMP response element binding** | |
| **HIF-1** | **Hypoxia inducible factor-1** | |
| **HIF-2** | **Hypoxia inducible factor-2** | |
| **CTCF** | **CCCTC-binding factor** | |


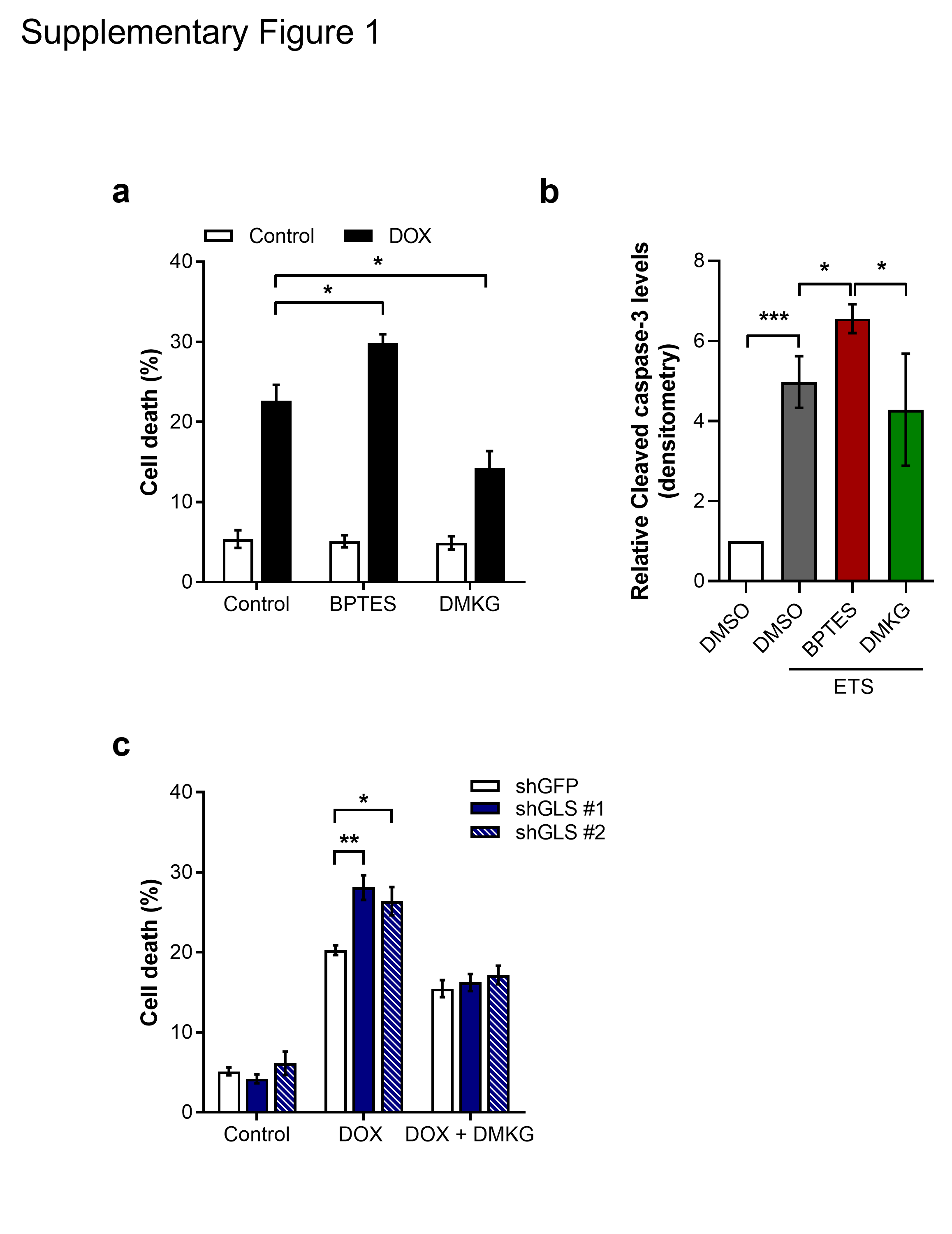

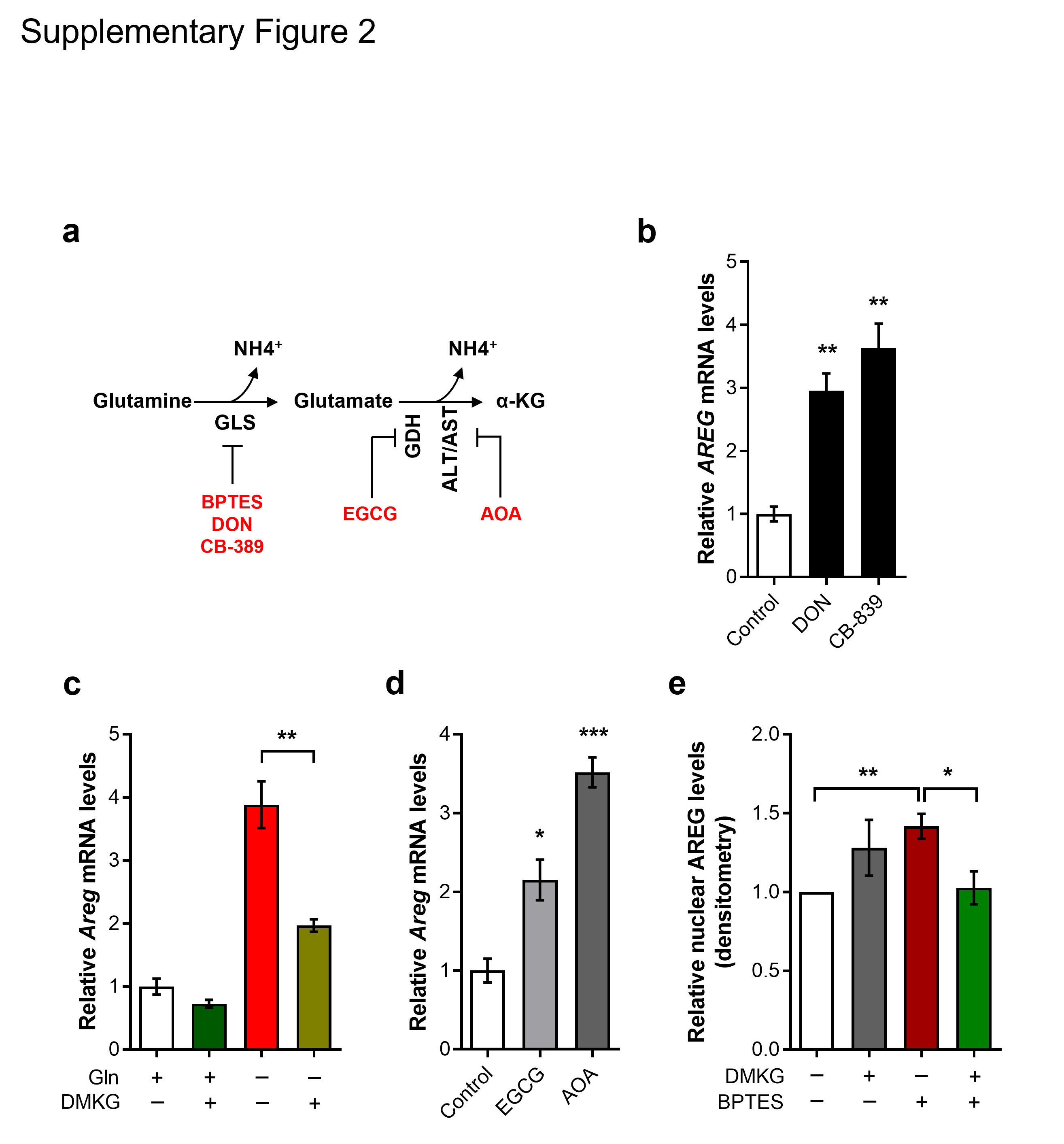

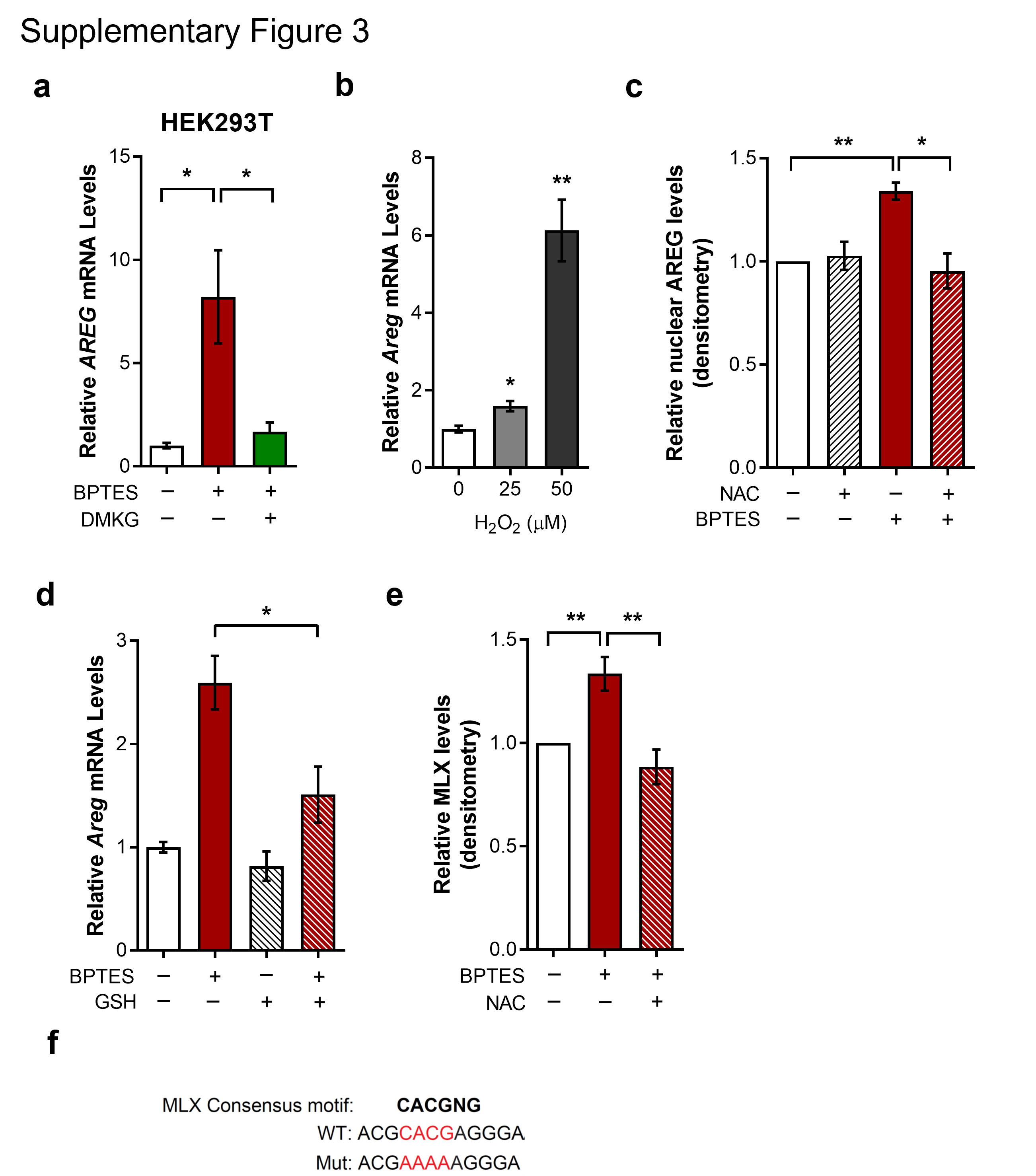

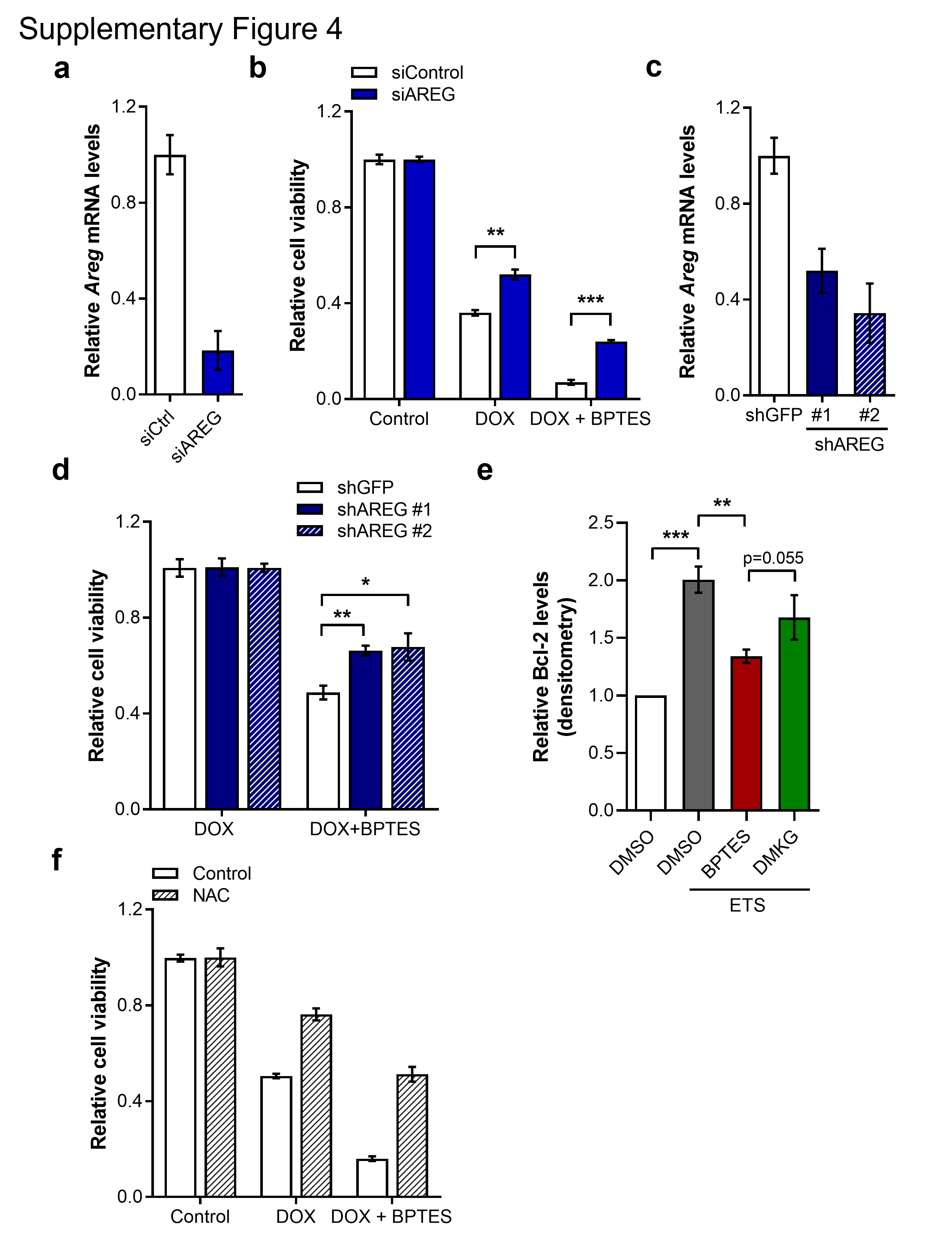

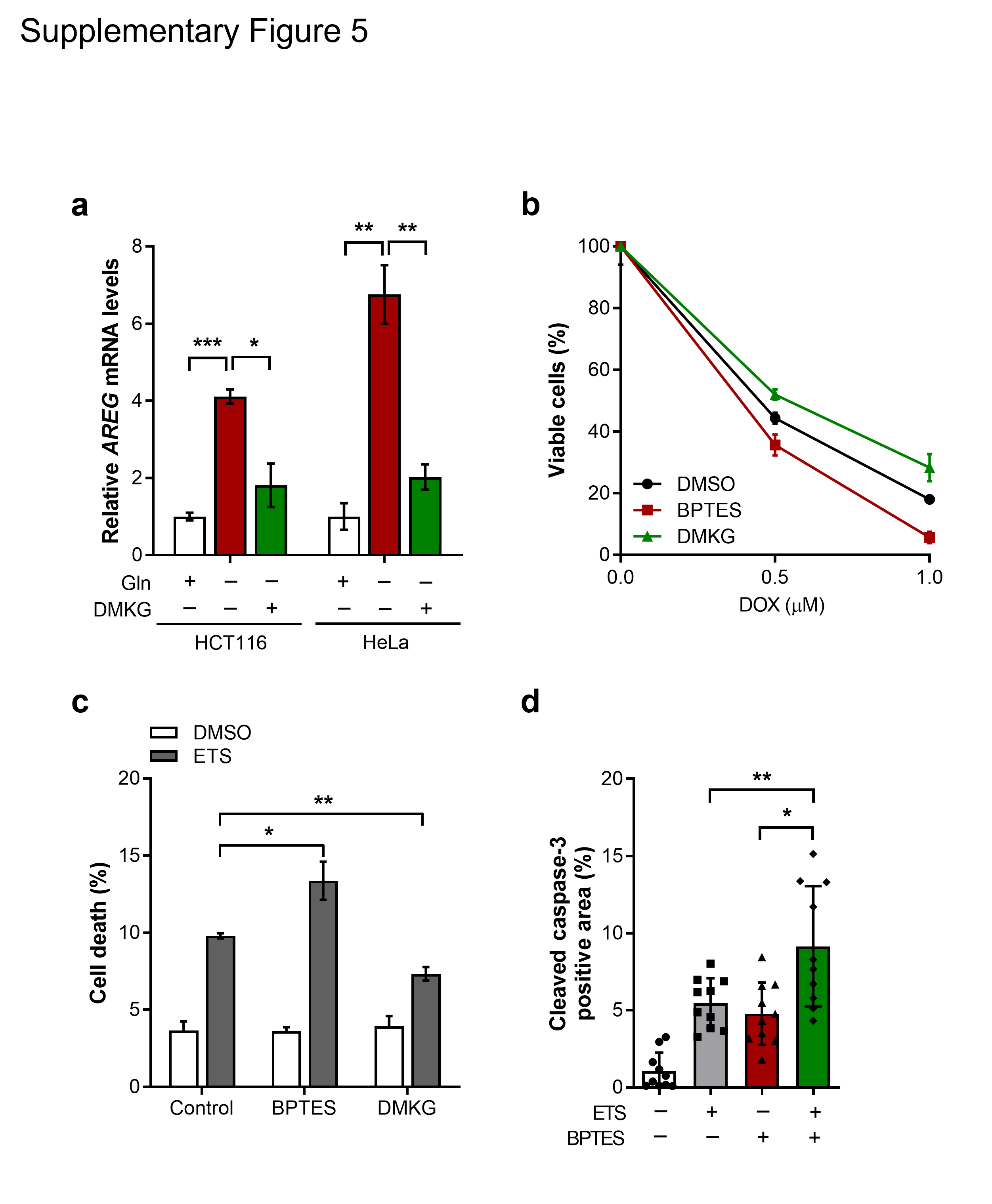

Supplement: Supplementary file 1 — supplementary information [file 41420_2021_792_MOESM1_ESM.docx]
